# Supplementary material for: In Vitro Study of Two Edible Polygonoideae Plants: Phenolic Profile, Cytotoxicity, and Modulation of Keap1-Nrf2 Gene Expression
Source: Foods. 2021 Apr 9;10(4):811. doi: 10.3390/foods10040811 (PMC8070220; doi:10.3390/foods10040811)
Supplement: Supplementary file 1 [file foods-10-00811-s001.pdf]

Supporting information for Subsection 2.2.

Five microlitres were injected into the system, and compounds were separated on Zorbax Eclipse XDB- C18 (50 mm × 4.6 mm, 1.8 μm) rapid resolution column held at 50˚C. Mobile phase was delivered at flow rate of 1 mL/min in gradient mode (0 min 30% B, 6 min 70% B, 9 min 100% B, 12 min 100% B, re-equilibration time 3 min). Eluted compounds were detected by MS, using the ion source parameters as follows: nebulization gas (N_2) pressure 40 psi, drying gas (N_2) flow 9 L/min and temperature 350 ˚C, capillary voltage 4 kV, negative polarity. Data were acquired in dynamic MRM mode, using the optimized compound specific parameters (retention time, precursor ion, product ion, fragmentor voltage, collision voltage) as it is published by Orčić et al. [27]. For all the compounds, peak areas were determined using Agilent MassHunter Workstation software—Qualitative Analysis (ver. B.06.00). Calibration curves were plotted and concentrations of samples calculated using the OriginLabs Origin Pro (ver. 8.0) software.

Supporting materials for Subsection 3.1.

Quantitative HPLC-MS-MS analysis:

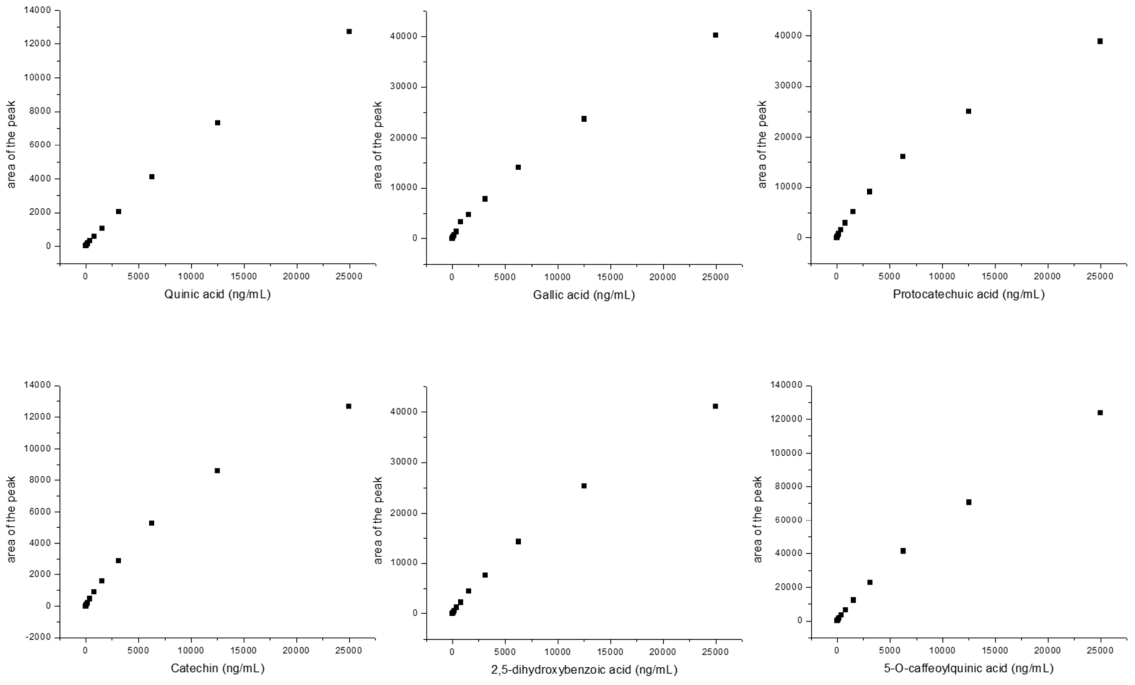

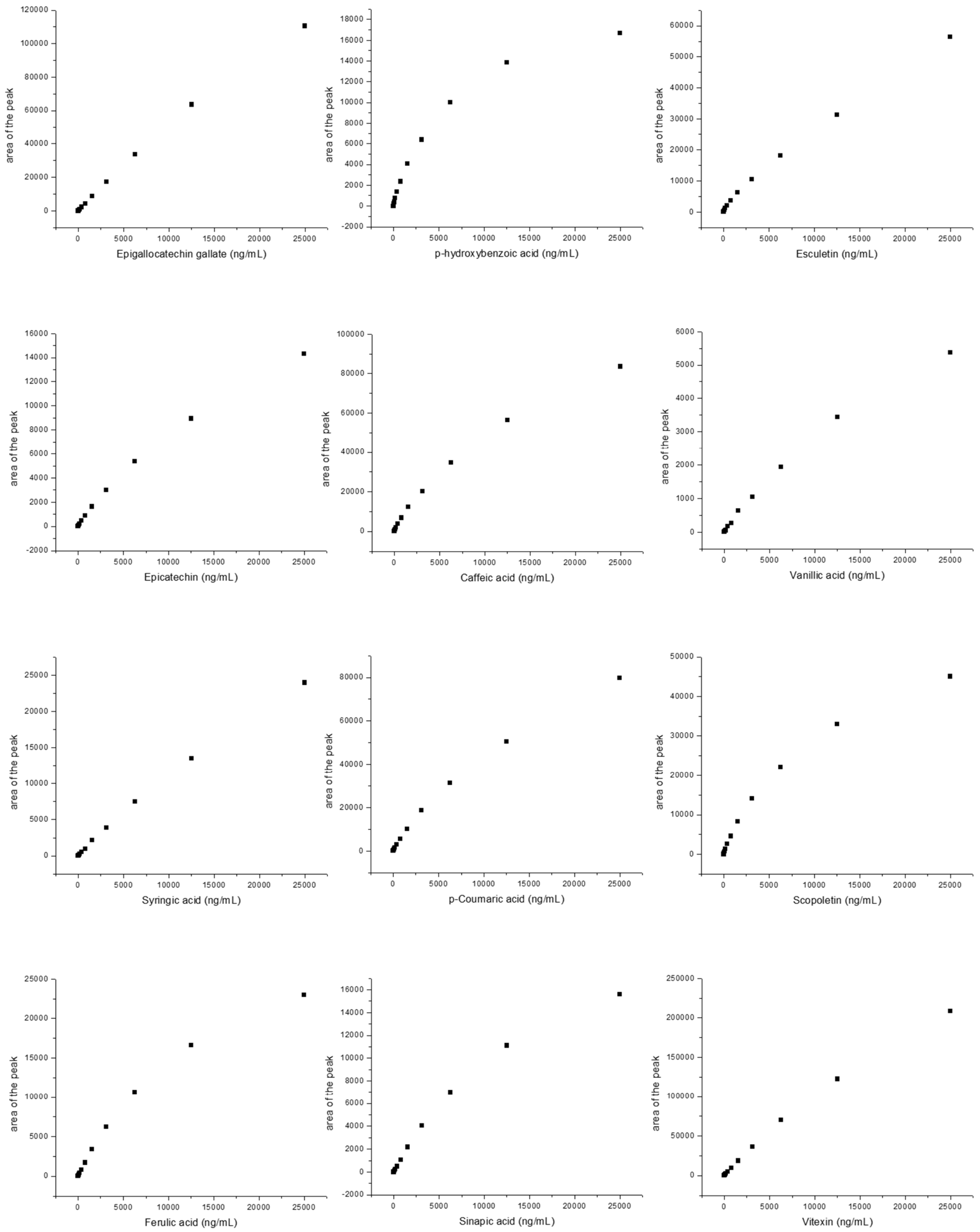

area of the peak
Epigallocatechin gallate (ng/mL)
p-hydroxybenzoic acid (ng/mL)
Esculetin (ng/mL)
Epicatechin (ng/mL)
Caffeic acid (ng/mL)
Vanillic acid (ng/mL)
Syringic acid (ng/mL)
p-Coumaric acid (ng/mL)
Scopoletin (ng/mL)
Ferulic acid (ng/mL)
Sinapic acid (ng/mL)
Vitexin (ng/mL)

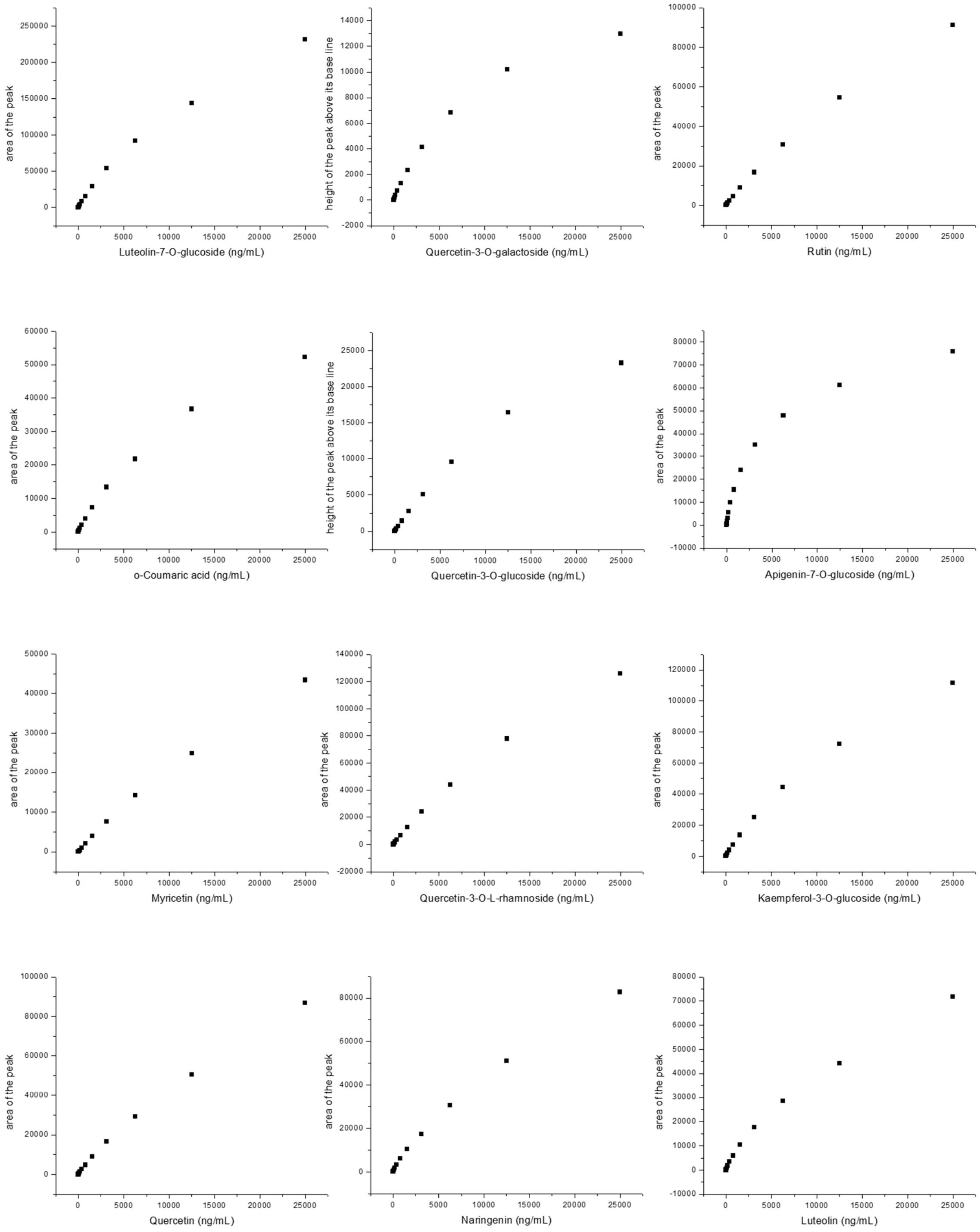

area of the peak
Luteolin-7-O-glucoside (ng/mL)
height of the peak above its base line
Quercetin-3-O-galactoside (ng/mL)
area of the peak
Rutin (ng/mL)
area of the peak
o-Coumaric acid (ng/mL)
height of the peak above its base line
Quercetin-3-O-glucoside (ng/mL)
area of the peak
Apigenin-7-O-glucoside (ng/mL)
area of the peak
Myricetin (ng/mL)
area of the peak
Quercetin-3-O-L-rhamnoside (ng/mL)
area of the peak
Kaempferol-3-O-glucoside (ng/mL)
area of the peak
Quercetin (ng/mL)
area of the peak
Naringenin (ng/mL)
area of the peak
Luteolin (ng/mL)

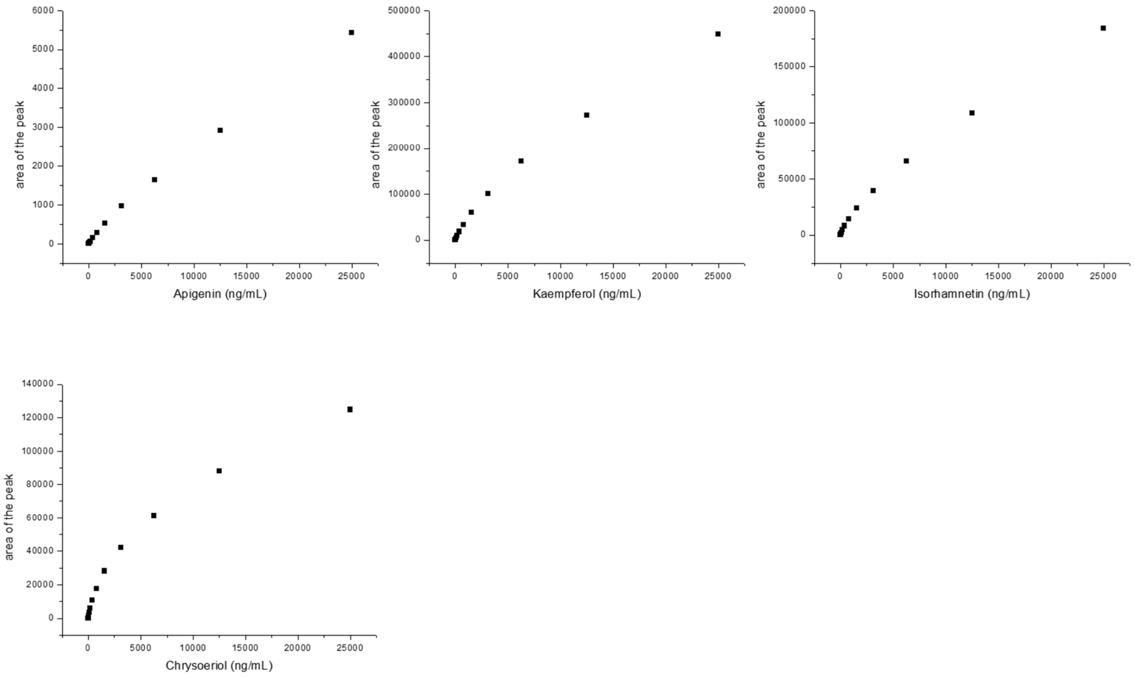

Figure S1 Calibration curves of standards (34 out of 45) which presence was detected in analysed extracts

Qualitative HPLC-DAD-MS analysis:

Table S1 Identification of the main compounds from *Polygonum aviculare* ethanol extracts (POA) by HPLC-DAD-MS

RT	**Labels on Fig. 1,2,3**	[M-H]-	[M+H]+		tentatively
min	**standards**	m/z	m/z	Standards	
0.729	**1**			Quinic acid	
0.841	**2**			Gallic acid	
1.267	**3**			Protocatechuic acid	
1.395	**4**			Catechin	
1.759	**6**			5-*O*-caffeoylquinic acid	
1.852	**7**			Epigallocatechin gallate	
2.259	**9**			Esculetin	
2.383	**11**			Caffeic acid	
2.421	**10**			Epicatechin	
**3.494**		**493-,317-**	**495+,319+**		Myricetin-3-*O*-glucuronide
**3.595**	**14**			*p*-Coumaric acid	
3.942	**19**			Vitexin	
4.068	**20**			Luteolin-7-*O*-glucoside	
**4.168**		**477-, 301-**	**479+,303+**		Quercetin-3-*O*-glucuronide
**4.219**	**21**			Quercetin-3-*O*-galactoside	
**4.246**	**22**			Quercetin-3-O-rutinoside	
**4.281**	**23**			Quercetin-3-*O*-glucoside	
**4.660**	**27**			Myricetin	
**4.672**		535-,317-	537+,319+		Myricetin-X''-*O*-acetyl-3-*O*-glucuronide
**4.714**		461-, 285-	463+,287+		Kaempferol-3-*O*-glucuronide
4.807	**28, 30**			Quercetin-3-*O*-L-rhamnoside, Kaempferol-3-*O*-glucoside	
**4.916**		491-,315-	493+,317+		Isorhamnetin-3-O-glucuronide
**5.02**		519-,301-	521+,303+		Quercetin-X''-*O*-acetyl-3-O-glucuronide
**5.21**		519-,301-	521+,303+		Quercetin-X''-*O*-acetyl-3-O-glucuronide
**5.501**	**35**			Quercetin	
**5.543**		503-,285-	505+,287+		Kaempferol-X''-*O*-acetyl-3-*O*-glucuronide
**5.629**		533-,315-	535+,317+		Isorhamnetin-X''-*O*-acetyl-3-*O*-glucuronide
**5.644**		503-,285-	505+,287+		Kaempferol-X''-*O*-acetyl-3-*O*-glucuronide
**5.714**	**38**			Luteolin	
**5.760**		533-,315-	535+,317+		Isorhamnetin-X''-*O*-acetyl-3-*O*-glucuronide
**5.838**		475-, 299-	477+,301+		Kaempferide-3-*O*-glucuronide
**6.159**	**40**			Kaempferol	

**6.272**	**39**			Apigenin	
**6.566**		517-,299-	519+,301+		Kaempferide-X''-*O*-acetyl-3-*O*-glucuronide

Table S2 Identification of the main compounds from *Persicaria amphibia* ethanol extracts (PEA) by HPLC-DAD-MS

RT	**Labels on Fig. 1,2,3**	[M-H]-	[M+H]+		tentatively
min	**standards**	m/z	m/z	Standards	
0.725	1			Quinic acid	
0.841	2			Gallic acid	
1.267	3			Protocatechuic acid	
1.391	4			Catechin	
1.875	7			Epigallocatechin gallate	
2.239	9			Esculetin	
2.387	11			Caffeic acid	
2.425	10			Epicatechin	
3.394	13				
3.487		493-,317-	495+,319+		Myricetin-3-*O*-glucuronide
3.564	14			p-Coumaric acid	
3.835	17				
4.168		477-, 301-	479+,303+		Quercetin-3-*O*-glucuronide
4.002	21			Quercetin-3-*O*-galactoside	
4.246	22			Quercetin-3-*O*-rutinoside	
4.273	23			Quercetin-3-*O*-glucoside	
4.664	27			Myricetin	
4.707		461-, 285-	463+,287+		Kaempferol-3-*O*-glucuronide
4.749	28, 30			Quercetin-3-*O*-L-rhamnoside, Kaempferol-3-O-glucoside	
5.652	37				
5.706	38			Luteolin	
6.152	40			Kaempferol	
6.264	39			Apigenin	

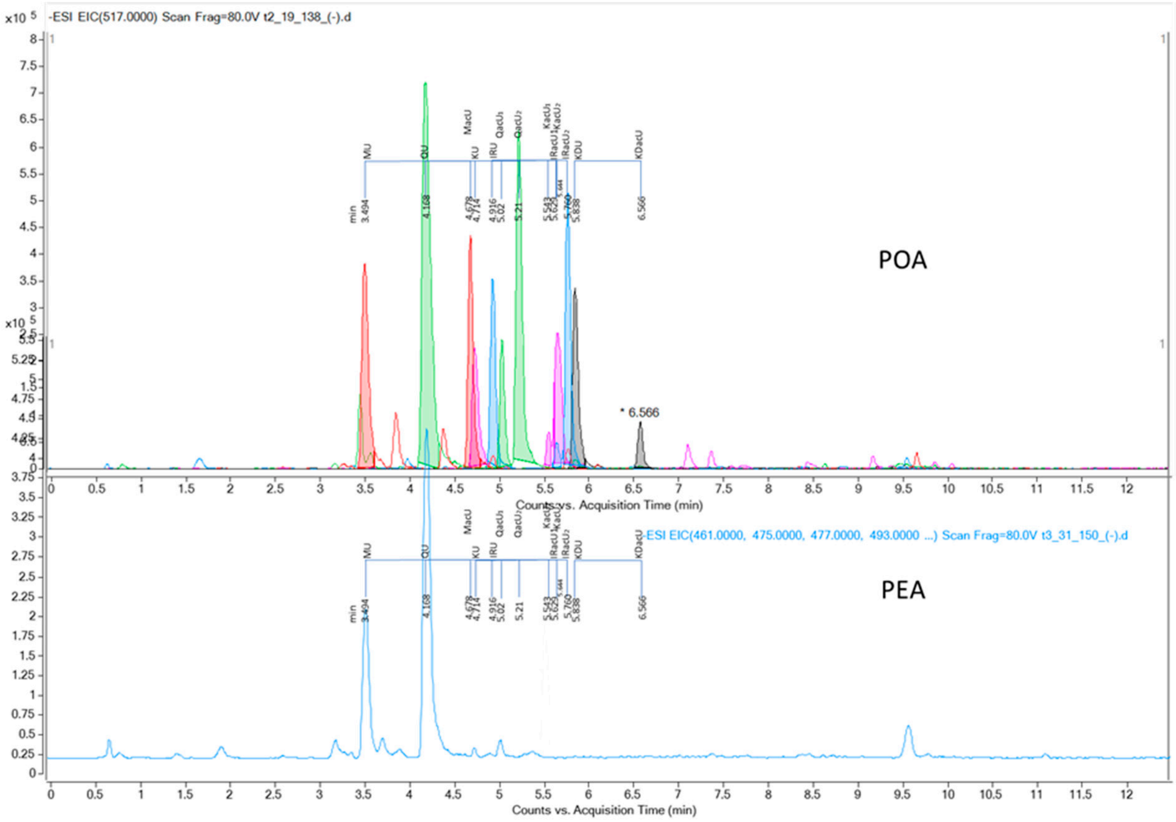

Figure S2 Extract Ion Chromatograms: -EIC: 493-,477-,461-,491-,535-,519-,475-,503-,533-,517-,317-,301-,285-,315-,299- indicating the significant differences in flavonol-glucronides composition in *P. aviculare* and *P. amphibia* species (labels explained in the paper).
